# Undrained capacity of circular shallow foundations on two-layer clays under combined VHMT loading

**DOI:** 10.1177/0309524X221142276

**Published:** 2023-01-12

**Authors:** Pengpeng He, Tim Newson

**Affiliations:** Department of Civil & Environmental Engineering, Western University, London, Canada

**Keywords:** Surficial crust, circular foundation, zero-tension interface, failure envelope, finite element analysis

## Abstract

Wind turbines are typically designed based on fatigue and serviceability limit states, but still require an accurate assessment of bearing capacity. Overconsolidated clay deposits in Canada often have a thin layer of crust with a relatively high undrained shear strength. However, existing bearing capacity design methods do not consider surficial crusts. This paper studies the undrained VHMT (vertical, horizontal, moment, and torsional) failure envelope of circular foundations founded on a surficial crust underlain by a uniform soil using finite element analysis. Crust correction factors have been introduced to account for the effects of the stiff layer on the vertical and moment capacities. The same forms of equation that are used for uniform soils, but with different parameters provide satisfactory fits for the failure envelopes for a soil with a crustal layer. An analytical expression for the 4-D VHMT failure envelope is derived, and an application of this method is provided.

## Introduction

Estimation of the bearing capacity of shallow foundations under combined loadings can be of great significance. This is particularly important for large onshore structures, such as wind turbines, transmission towers and masts, due to their complex environmental loadings. The majority of offshore foundations are circular or close to circular in form. For wind turbines, apart from the vertical load due to its self-weight, the horizontal load induced by wind is also substantial and a large structural height further leads to a significant moment load on the foundation.

Traditionally, this type of design is based on classical solutions for the uniaxial vertical bearing capacity of shallow foundations using the superposition principle (Terzaghi, 1951). The effects of load inclination and eccentricity are incorporated by introducing a load inclination factor and an effective foundation area (e.g. DNV, GL, 2016). Since load inclination and eccentricity effects are separately considered, this semi-empirical modification of the conventional theory may sometimes be insufficiently accurate for practical design (Gourvenec, 2007). This approach is still common for onshore shallow wind turbine foundation design.

The failure envelope method has been recommended as an alternative to conventional theories in some geotechnical design guidelines (particularly those focused on offshore geotechnics), such as API (2011) and International Standards Organisation (ISO) (2016), due to the load interaction effect between various load components (i.e. combined vertical, horizontal, moment, and torsional loads) being explicitly incorporated (Shen et al., 2017). Failure envelopes for different types of foundations (e.g. strip (Bransby and Randolph, 1998; Fathipour et al., 2022), rectangular (Gourvenec and Randolph, 2003) and circular (Suryasentana et al., 2021) foundations), homogeneous (Taiebat and Carter, 2010) or non-homogeneous (He and Newson, 2022b; Wu et al., 2022) soils, and zero-tension (Shen et al., 2016) or unlimited-tension (Gourvenec and Randolph, 2003) interface conditions have been previously investigated under undrained soil conditions. These studies have been primarily confined to a single layer soil with a uniform or linearly increasing soil undrained shear strength profile. However, an onshore clay deposit often has a thin layer of stiff crust with a relatively high undrained shear strength developed from weathering, desiccation, and chemical process (Lutenegger, 1995). The shear strength of the upper crustal layer can be more than 10 times that of the underlying clay (Lee and Park, 1999). Nakase et al. (1978) and Sagaseta and Arroyo (1982) have demonstrated that the undrained shear strength profile of the crust strongly affects the stability analysis of shallow foundations and embankments because a substantial portion of the failure surface under the structure can be located within the crust. Understanding the effect of a surficial crust on the bearing capacity of shallow foundations is therefore important for optimal foundation design. However, existing design standards (e.g. DNV, GL, 2016) predict the bearing capacity of shallow foundations under idealized soil conditions without considering the existence of the surficial crust.

Studies available in the literature that account for the effect of a high strength surficial layer on foundation bearing capacity are still sparse. Merifield et al. (1999) evaluated the undrained bearing capacity of a centrally, vertically loaded surface strip foundation on a two-layer clay deposit using numerical upper and lower bound analysis. Recently, the same cases for square and circular foundations were further investigated by Merifield and Nguyen (2006) using finite element (FE) analysis. However, this considers only vertical bearing capacity in the absence of horizontal, moment and torsional loads. Park et al. (2010) determined the bearing capacity factor *N*_c_ for strip and circular foundations resting on a non-homogeneous crust overlying a uniform soil, but the failure envelopes under combined loadings were not addressed. Feng et al. (2015) studied the failure envelope of a rectangular foundation founded on a crustal layer overlying a normally consolidated clay using an unlimited-tension soil-foundation interface. However, unlike skirted shallow foundations of offshore structures, onshore shallow foundations can uplift and separate from the soil under a large overturning moment because the soil-foundation interface cannot provide tensile resistance (i.e. zero-tension interface). Xia et al. (2022) explored the VHM envelope of bucket foundations in a two-layer clay, but the soil is soft-over-stiff rather than stiff-over-soft.

The object of this paper is to investigate the VHMT failure envelope of circular foundations founded on a surficial crust underlain by a uniform soil under a zero-tension interface condition using finite element analysis. Undrained soil conditions have been considered for both the crustal layer and the underlying soil. The effects of the surficial crust on the failure envelope have been studied. An analytical expression for the 4-D VHMT failure envelope is also derived in this paper. The derived 4-D failure envelope has been briefly summarized in a technical note by the authors (i.e. He and Newson, 2022a), but the detailed procedures to derive the envelope were not provided. As a complement, this paper presents the analysis of deriving the final 4-D envelope in more detail and, an example of the use of this method for design is also provided.

## Method—finite element analysis

### Material models and interface conditions

A linear elastic perfectly plastic constitutive relationship with a Mohr-Coulomb (M-C) failure criterion was used to model the soil behavior. For undrained soil conditions, the M-C criterion degenerates to the Tresca criterion, which is defined by three soil parameters: the undrained Young’s modulus, *E*_u_, Poisson’s ratio, *µ*, and undrained shear strength, *s*_u_.

**Figure 1.**
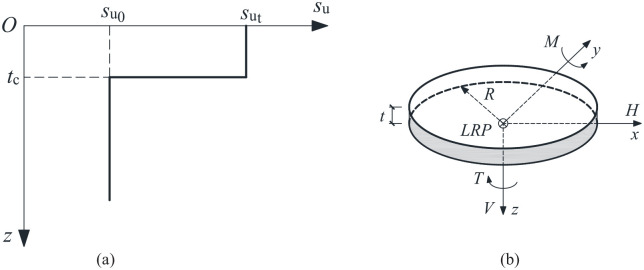
Soil profile and sign conventions: (a) soil profile with a surficial crust and (b) sign conventions.

As shown in Figure 1a, two fundamental parameters of the surficial crust layer may affect the bearing capacity of a surface foundation on a crusted soil: the average crust undrained shear strength, *s*_ut_, and the crust thickness, *t*_c_. As suggested by Lee and Park (1999), in some cases the average shear strength of the upper crust layer can be more than 10 times that of the underlying clay. In this paper, the ratio, *s*_u0_/*s*_ut_ (*s*_u0_ refers to the undrained shear strength of the underlying soil), has been varied from 0.2 to 1.0 (i.e. 0.2, 0.4, 0.6, 0.8, and 1.0), to represent a typical range of soil profiles. This approach addresses cases of strong-over-soft clays adopted by Merifield et al. (1999). The special case of s_u0_/s_ut_ = 1 relates a homogeneous soil. As shown by Bjerrum (1973), the thickness of a crust layer usually ranges from 1 to 8 m depending upon the hydrogeology (i.e. well-drained versus poorly drained). In eastern Canada, the crust thickness is generally 1–5 m and is often of the order of 3 m (Lefebvre et al., 1987). Typically, the diameter (*D*) of an onshore wind turbine foundation is very large (>15 m), and these foundations are becoming larger with the increase of power output and tower height. The foundation diameter used in this paper is 19 m, representing the typical dimension for current onshore wind turbines in Canada. Therefore, this study has considered models with *t*_c_/*D* ranging from 0.1 to 0.3 (i.e. 0.1, 0.2, and 0.3) to span most cases of practical interest. This range also covers that used by Feng et al. (2015).

As an initial study, the undrained shear strength of the underlying soil (i.e. *s*_u0_) is considered to be constant with depth for simplicity. In the analysis, *s*_u0_ was held constant at 100 kPa which is a typical value for the underlying soil in Ontario Canada. The undrained soil Poisson’s was taken to be 0.495. Since the soil Young’s modulus, *E*_u_, affects only the evolution of the load-displacement relationship but not the calculated collapse loads (Yun and Bransby, 2007), a sufficiently large *E*_u_/*s*_u0_ ratio equal to 10,000 was selected to minimize mesh distortion (Abyaneh et al., 2015). The foundation was assumed to be a rigid body, and a load reference point (LRP) was used to apply prescribed displacements or loads, located at the bottom center of the foundation.

Similar to Shen et al. (2016), the FE model considered a zero-tension, rough base that allows separation of the foundation from the soil. The zero-tension rough base can be modeled using a Coulomb model with a friction coefficient of 20 (Shen et al., 2016).

### Geometry and mesh

The analysis in this paper was conducted using the finite element software ABAQUS (Dassault Systèmes, 2016). To avoid the effects of model boundaries on the development of the failure mechanisms, the mesh length, *L*, and mesh height, *H*, were taken as 120 and 50 m, respectively, following the recommendation of Deshpande (2016).

**Figure 2.**
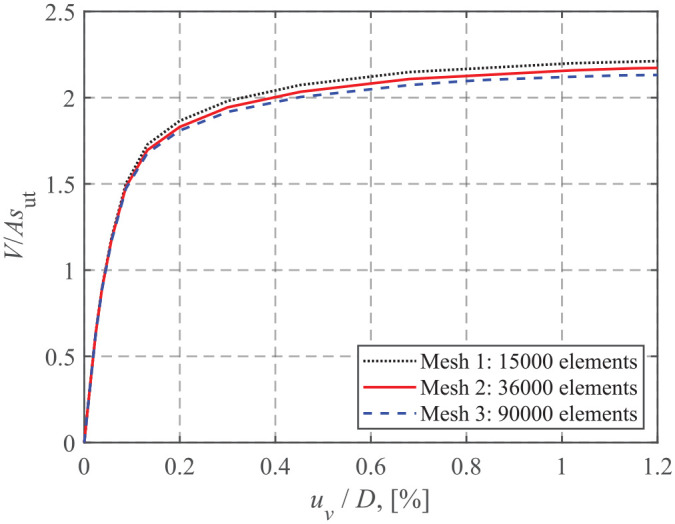
Mesh convergence study for the case of s_u0_/s_ut_ = 0.2 and t_c_/D = 0.3.

**Figure 3.**
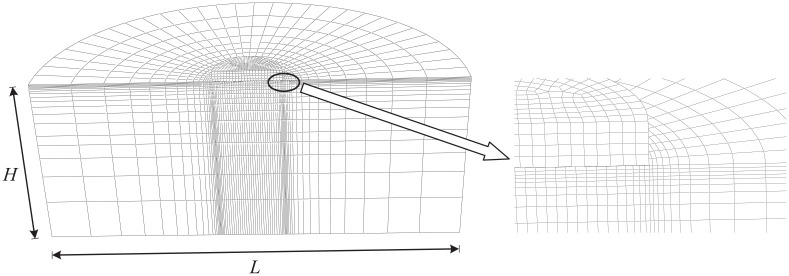
Half-view of the FE mesh.

A mesh convergence study was carried out for a number of cases. A typical outcome is shown in Figure 2. The model solution with Mesh 3 takes about six times longer than that using Mesh 2, but the difference between the ultimate vertical capacities using Meshes 2 and 3 is only around 2%. Thus, this study adopted Mesh 2 in the following analyses. Figure 3 shows the half-model of the 3-D model using Mesh 2 (He and Newson, 2022a). This mesh was composed of approximately 36,000 8-noded brick elements (i.e. first-order, ABAQUS C3D8R with reduced integration and hourglass control). Following Gourvenec and Randolph (2003), the soil regions in the vicinity of the foundation edge and the horizontal thin soil layer close to the interface were carefully refined to capture the intense stress concentration close to the foundation edge and the large plastic shear strains at the interface. The cylindrical circumference of the soil mass was constrained to prevent out-of-plane translations, and the bottom of the soil mass was fixed in the three orthogonal directions.

### Sign conventions and loading paths

The sign conventions for the loads are shown in Figure 1b. The horizontal and moment loads were considered to be in the same plane.

**Figure 4.**
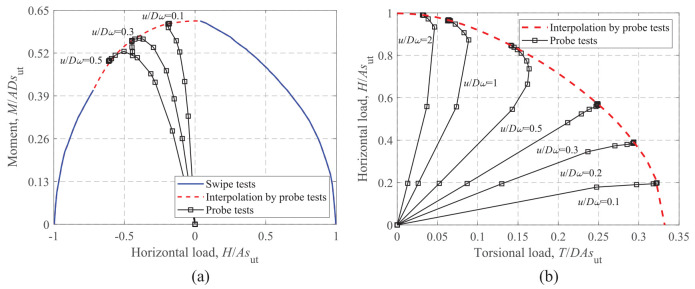
*M*-*H* and *H*-*T* failure envelopes for a uniform soil at V/V_ult_ = 0.50: (a) *M*-*H* and (b) *H*-*T.*

Probe tests and swipe tests were used to track the failure envelopes under different loading conditions. One probe test provides only a single point on a failure envelope. A swipe test is able to track the whole or a portion of a failure envelope. Refer to He and Newson (2022b) for more details of probe tests and swipe tests. Two typical failure envelopes obtained using the swipe and probe tests are shown in Figure 4.

## Finite element results

**Figure 5.**
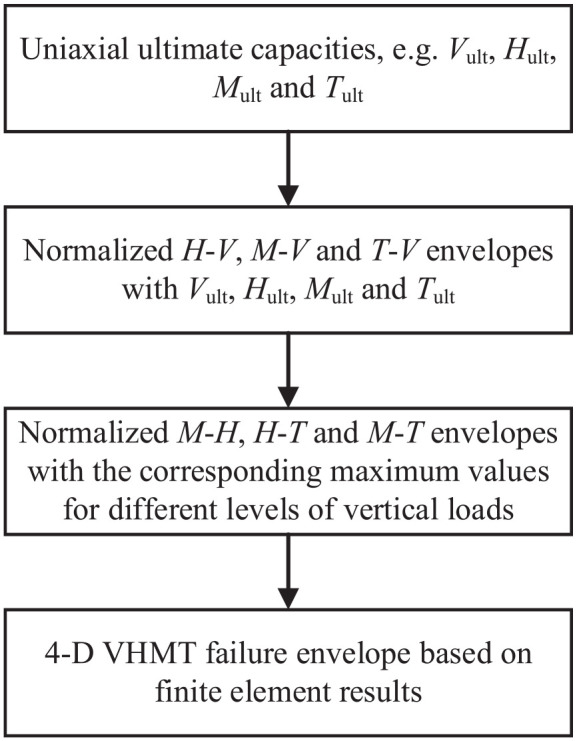
Overall strategy for the finite element analysis.

As shown in Figure 5, the overall strategy for assessing the undrained bearing capacity of a shallow foundation under combined VHMT loads is decomposed into three steps: (i) determining uniaxial ultimate capacities, for example *V*_ult_, *H*_ult_, *M*_ult_, and *T*_ult_; (ii) normalizing *H*-*V*, *M*-*V*, and *T*-*V* failure envelopes with the corresponding uniaxial ultimate capacities; and (iii) normalizing *M*-*H*, *H*-*T*, and *M*-*T* failure envelopes with the corresponding maximum values. Using the results from the above steps, the failure envelope under combined VHMT loadings can be derived.

### Pure uniaxial capacities

The ultimate loads for vertical, horizontal and torsional modes are referred to as the corresponding uniaxial load-carrying capacities in the absence of the other loading modes. Note that the torsional and moment loads on an onshore wind turbine foundation result from the effects of horizontal loading (i.e. horizontal load non-coplanarity and tower height). As a foundation with a zero-tension interface cannot resist any moment loading without vertical loads, the ultimate moment capacity is represented by the maximum moment load under vertical loading only. Analyses have shown that the uniaxial horizontal and torsional capacities of a surface foundation are purely related to the undrained shear strength of the surface soil: $H_{\mathrm{ult}}=As_{\mathrm{ut}}$ and $T_{\mathrm{ult}}=\mathrm{AD}s_{\mathrm{ut}}/3$ , which has also been confirmed by previous studies (e.g. Finnie and Morgan, 2004; He and Newson, 2020). Therefore, only the vertical and moment capacities have been investigated in this section.

For validation purposes, the current results for a single-layer clay are compared to those from previous studies (Shen et al., 2016; Taiebat and Carter, 2010) in Table 1. It can be seen that the comparison is satisfactory.

**Table 1.** Uniaxial bearing capacity factors for a single-layer clay.

Bearing capacity factor	$V_{\mathrm{ult}}/\left(As_{\mathrm{ut}}\right)$	$H_{\mathrm{ult}}/\left(As_{\mathrm{ut}}\right)$	$M_{\mathrm{ult}}/\left(\mathrm{AD}s_{\mathrm{ut}}\right)$	$T_{\mathrm{ult}}/\left(\mathrm{AD}s_{\mathrm{ut}}\right)$
Current study	6.00	1.00	0.62	0.33
Taiebat and Carter (2010)	6.17	1.00	0.62	–
Shen et al. (2016)	5.87	1.02	0.61	–

The crust correction factor, *s_cr_*, defined as the ratio of the dimensionless capacity for a crusted soil to that of a uniform soil, is introduced to characterize the effect of a surficial crust:

(1) $$s_{\mathrm{cr}}=\frac{N_{s_{u0}/s_{\mathrm{ut}}}}{N_{s_{u0}/s_{\mathrm{ut}}=1}}$$

where $N_{s_{u0}/s_{\mathrm{ut}}}^{v}=V_{\mathrm{ult}}/\left(As_{\mathrm{ut}}\right)$ for the vertical capacity and $N_{s_{u0}/s_{\mathrm{ut}}}^{m}=M_{\mathrm{ult}}/\left(\mathrm{AD}s_{\mathrm{ut}}\right)$ for the moment capacity.

**Figure 6.**
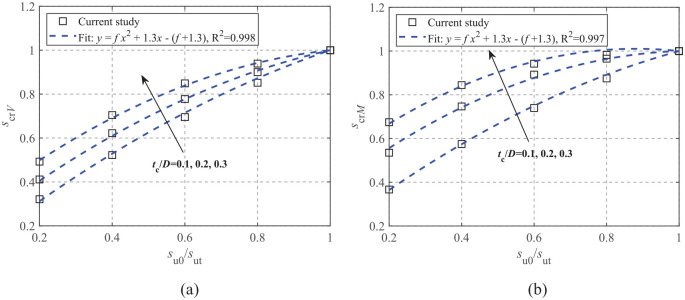
Crust correction factors of uniaxial capacities (He and Newson, 2022a, 2022b): (a) *V*_ult_ and (b) *M*_ult_.

Note that the crust correction factors for horizontal and torsional loading modes remain at unity. The variations of *s_crV_* and *s_crM_* with regard to *s*_u0_/*s*_ut_ and *t*_c_/*D* are shown in Figure 6 (He and Newson, 2022a). The vertical and moment factors significantly increase with *s*_u0_/*s*_ut_ and gradually converge to unity as *s*_u0_/*s*_ut_ approaches unity. A quadratic polynomial equation with respect to *s*_u0_/*s*_ut_ is proposed to estimate the relationships:

(2) $$s_{\mathrm{cr}}=f\cdot {\left(\frac{s_{u0}}{s_{\mathrm{ut}}}\right)}^{2}+1.3\left(\frac{s_{u0}}{s_{\mathrm{ut}}}\right)-\left[\;f+0.3\right]$$

The coefficient, *f*, is a function of *t*_c_/*D*, defined as $f\left(t_{c}/D\right)=-0.97t_{c}/D-0.27$ for the vertical capacity and $f\left(t_{c}/D\right)=\frac{-1.18t_{c}/D}{t_{c}/D+0.18}$ for the moment capacity. As seen in Figure 6, the curve fitting is in close agreement with the FE results.

**Figure 7.**
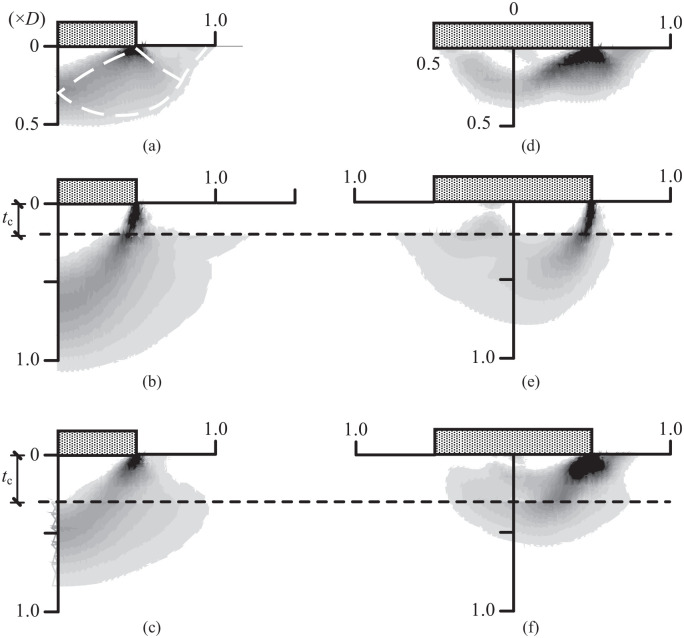
Collapse mechanisms at *V*_ult_ and *M*_ult_: (a) *V*_ult_: Uniform soil, (b) *V*_ult_: s_u0_/s_ut_ = 0.2, t_c_/D = 0.2, (c) *V*_ult_: s_u0_/s_ut_ = 0.6, t_c_/D = 0.3, (d) *M*_ult_: Uniform soil, (e) *M*_ult_: s_u0_/s_ut_ = 0.2, t_c_/D = 0.2, and (f) *M*_ult_: s_u0_/s_ut_ = 0.6, t_c_/D = 0.3.

To better understand the effects of *s*_u0_/*s*_ut_ and *t*_c_/*D* on the collapse mechanism at *V*_ult_ and *M*_ult_, Figure 7 shows the contours of the maximum plastic shear strain increment for a uniform soil (Figure 7a and d), the case of s_u0_/s_ut_ = 0.2 and t_c_/D = 0.2 (Figure 7b and e), and the case of s_u0_/s_ut_ = 0.6 and t_c_/D = 0.3 (Figure 7c and f). The geometry of the collapse mechanism can be effectively visualized using the maximum plastic shear strain increment (Loukidis et al., 2008):

(3) $$\Delta \gamma_{\max}^{p}=\sqrt{{\left(\Delta \varepsilon_{\mathrm{xx}}^{p}-\Delta \varepsilon_{\mathrm{yy}}^{p}\right)}^{2}+{\left(\Delta \varepsilon_{\mathrm{xx}}^{p}-\Delta \varepsilon_{\mathrm{zz}}^{p}\right)}^{2}+{\left(\Delta \varepsilon_{\mathrm{yy}}^{p}-\Delta \varepsilon_{\mathrm{zz}}^{p}\right)}^{2}+{\left(\Delta \gamma_{\mathrm{xy}}^{p}\right)}^{2}+{\left(\Delta \gamma_{\mathrm{xz}}^{p}\right)}^{2}+{\left(\Delta \gamma_{\mathrm{yz}}^{p}\right)}^{2}}$$

where $\Delta \varepsilon_{\mathrm{xx}}^{p}$ , $\Delta \varepsilon_{\mathrm{yy}}^{p}$ , $\Delta \varepsilon_{\mathrm{zz}}^{p}$ , $\Delta \gamma_{\mathrm{xy}}^{p}$ , $\Delta \gamma_{\mathrm{xz}}^{p}$ and $\Delta \gamma_{\mathrm{yz}}^{p}$ are the plastic normal and shear strain increments in Cartesian coordinates.

Figure 7a–c shows the failure mechanisms at *V*_ult_. As shown in Figure 7a, the failure mechanism for a uniform soil compares well to that of general shear failure (white dashed lines) found from the method of characteristics (Martin, 2003). The comparison between Figure 7a–c shows that the depth of the active triangular zone remains almost the same, although the active triangular zone for the case of s_u0_/s_ut_ = 0.2 and t_c_/D = 0.2 is slightly curved. However, the shear fan zone for the case of s_u0_/s_ut_ = 0.2 and t_c_/D = 0.2 extends to a depth of 1*D*, but ends at the base of the crust. In addition, the case of s_u0_/s_ut_ = 0.2 and t_c_/D = 0.2 has no passive zone close to the ground surface, indicating a local shear failure mode. This is because the relatively strong top crust acts as rigid column that restricts both upward and lateral deformations within the crustal layer, while this restriction in turn increases the depth of the failure zone within the bottom layer. A partial shear failure mechanism also appears to be initiating in the lower layer for the two crusted cases. This phenomenon was also observed for both square and circular foundations by Merifield and Nguyen (2006). Figure 7c shows that the failure mechanism at *V*_ult_ for the case of s_u0_/s_ut_ = 0.6 and t_c_/D = 0.3 lies in between Figure 7a and b, since this case has a moderately strong surficial crust.

The collapse mechanisms at *M*_ult_ (at V/V_ult_ = 0.50) are shown in Figure 7d–f. For the uniform soil, a combined scoop-wedge mechanism is observed (similar to that found by Bransby and Randolph, 1998). Compared to the uniform soil case, the failure zone for the case of s_u0_/s_ut_ = 0.2 and t_c_/D = 0.2 is primarily confined to the underlying layer, and the crust behaves as a rigid column due to the relatively high strength. A wedge mechanism still exists on the right hand side of the foundation, but the scoop seems to have been suppressed. Moreover, the failure pattern for the case of s_u0_/s_ut_ = 0.6 and t_c_/D = 0.3 lies in between the other two cases. Therefore, the main effect of a crustal layer on the collapse zone is the suppression of the surface failure and the scoop portion of the mechanisms, and an increase of the depth of the failure zone within the underlying layer.

### Horizontal-vertical loading

**Figure 8.**
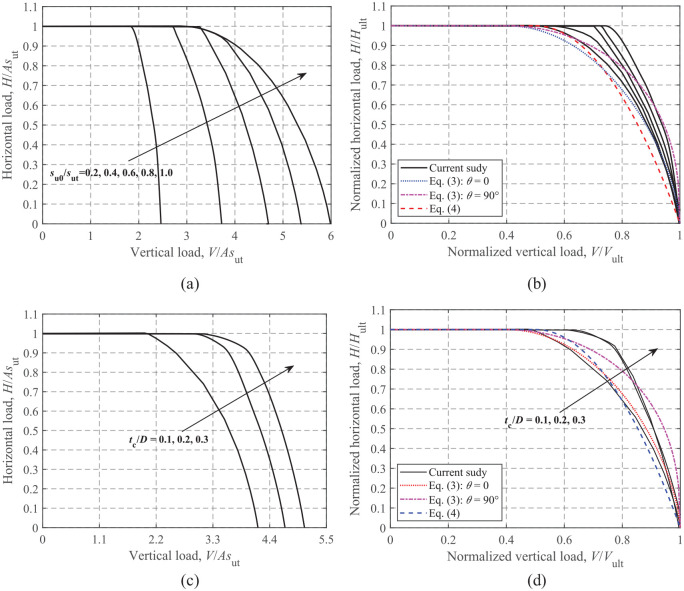
*H*-*V* failure envelopes: (a) dimensionless, s_u0_/s_ut_ = 0.2–1.0 and t_c_/D = 0.2, (b) normalized, s_u0_/s_ut_ = 0.2–1.0 and t_c_/D = 0.2, (c) dimensionless, s_u0_/s_ut_ = 0.6 and t_c_/D = 0.1–0.3, and (d) normalized, s_u0_/*s*_ut_ = 0.6 and t_c_/D = 0.1–0.3.

Figure 8a and b shows the effects of *s*_u0_/*s*_ut_ on the dimensionless and normalized *H-V* failure envelopes using the cases of s_u0_/s_ut_ = 0.2–1.0 and t_c_/D = 0.2. The dimensionless envelopes shown in Figure 8a exhibit an expansion of the curves with increasing *s*_u0_/*s*_ut_. Note also that a stiffer *H-V* failure envelope is observed for a smaller value of *s*_u0_/*s*_ut_, that is a lower *s*_u0_/*s*_ut_ ratio corresponds to a larger normalized *H-V* envelope, as shown in Figure 8b. For rectangular foundations on a soil with a crust using an unlimited-tension interface, Feng et al. (2015) proposed an analytical equation (see equation (4)) as a function of the loading angle, *θ*. The curves with *θ* = 0 and 90° are presented in Figure 8b. In addition, a curve fit to Green’s exact solution (equation (5)) is also widely used to describe *H-V* envelopes. The comparison shows that equation (4) with *θ* = 0 and equation (5) provide conservative predictions of the FE results. For simplicity, equation (5) is considered to model the current *H-V* failure envelope by ignoring its slight dependence on *s*_u0_/*s*_ut_.

(4) $$\begin{array}{c} V/V_{\mathrm{ult}}=0.4+0.6\sqrt{1-{\left(H/H_{\mathrm{ult}}\right)}^{2.5-\cos^{2}\theta }},\;\mathrm{for}\;V/V_{\mathrm{ult}}\geq 0.40\\ H/H_{\mathrm{ult}}=1,\;\mathrm{for}\;V/V_{\mathrm{ult}}<0.40 \end{array}$$

(5) $$\begin{array}{c} V/V_{\mathrm{ult}}=0.5+0.5\sqrt{1-H/H_{\mathrm{ult}}},\;\mathrm{for}\;V/V_{\mathrm{ult}}\geq 0.50\\ H/H_{\mathrm{ult}}=1,\;\mathrm{for}\;V/V_{\mathrm{ult}}<0.50 \end{array}$$

The cases of s_u0_/s_ut_ = 0.6 and t_c_/D = 0.1–0.3 are presented in Figure 8c and d to show the effect of the normalized crust thickness *t*_c_/*D* on the *H-V* failure envelopes. As shown in Figure 8c, the envelope size increases with *t*_c_/*D*, but the rate of increase appears to gradually decrease. It can also be seen that equation (5) also provides a relatively conservative evaluation of the normalized curves, although dispersion of the curves caused by *t*_c_/*D* can be observed.

### Moment-vertical loading

**Figure 9.**
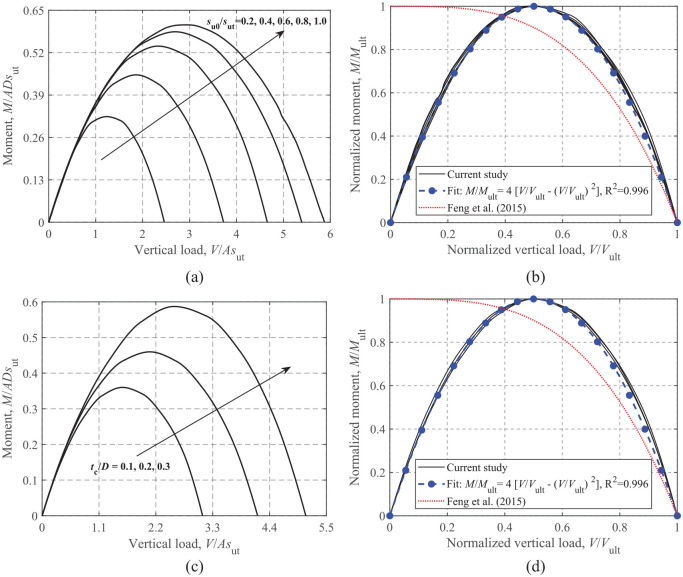
*M*-*V* failure envelopes: (a) dimensionless, s_u0_/*s*_ut_ = 0.2–1.0 and t_c_/D = 0.2, (b) normalized, s_u0_/*s*_ut_ = 0.2–1.0 and t_c_/D = 0.2, (c) dimensionless, s_u0_/*s*_ut_ = 0.6 and t_c_/D = 0.1–0.3, and (d) normalized, s_u0_/*s*_ut_ = 0.6 and t_c_/D = 0.1–0.3.

Figure 9 shows the effects of *s*_u0_/*s*_ut_ and *t*_c_/*D* on the *M-V* failure envelopes. A significant expansion of the failure loci with increasing *s*_u0_/*s*_ut_ and *t*_c_/*D* is observed in Figure 9a and c, although the rate of expansion gradually decreases with *s*_u0_/*s*_ut_. The failure envelopes in terms of loads normalized by their ultimate values in Figure 9b and d fall in a very tight band, with the shape following the parabolic function given by equation (6).

(6) $$M/M_{\mathrm{ult}}=4\left[V/V_{\mathrm{ult}}-{\left(V/V_{\mathrm{ult}}\right)}^{2}\right]$$

The equation proposed by Feng et al. (2015) for rectangular foundations on a soil with a crust under an unlimited-tension interface is also incorporated in Figure 9, which differs considerably from the current FE results due to the unlimited-tension interface.

### Torsion-vertical loading

**Figure 10.**
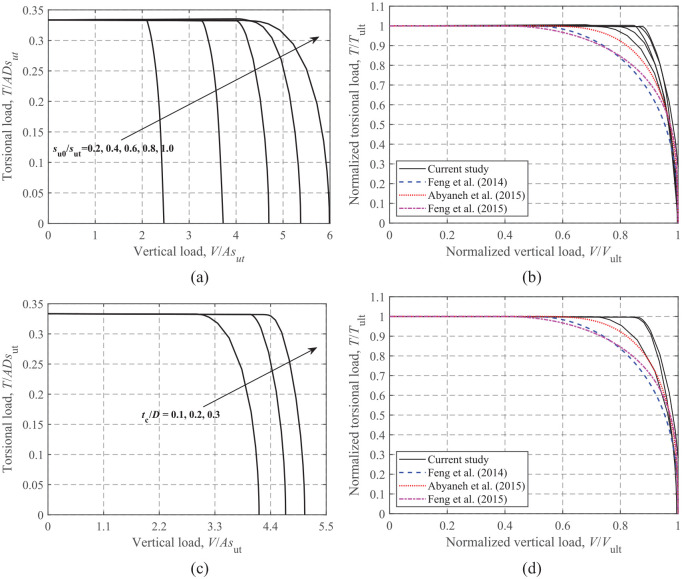
*T*-*V* failure envelopes: (a) dimensionless, s_u0_/*s*_ut_ = 0.2–1.0 and t_c_/D = 0.2, (b) normalized, s_u0_/*s*_ut_ = 0.2–1.0 and t_c_/D = 0.2, (c) dimensionless, s_u0_/*s*_ut_ = 0.6 and t_c_/D = 0.1–0.3, and (d) normalized, s_u0_/*s*_ut_ = 0.6 and t_c_/D = 0.1–0.3.

Figure 10 shows the effects of *s*_u0_/*s*_ut_ and *t*_c_/*D* on the *T-V* failure envelopes. Similar to the horizontal capacity, the torsional capacity is also determined only by the surface soil strength, therefore, the *T-V* failure envelopes share similar features with increasing *s*_u0_/*s*_ut_ and *t*_c_/*D* to the *H-V* envelopes. The analytical equations proposed by Feng et al. (2014) (see equation (7)), Abyaneh et al. (2015) (see equation (8)) and Feng et al. (2015) (see equation (9)) are compared to the FE results in Figure 10b and 10d. The comparison shows that the formula of Abyaneh et al. (2015) gives reasonable and conservative predictions for the normalized failure envelope.

(7) $$\begin{array}{c} T/T_{\mathrm{ult}}={\left[1-4{\left(V/V_{\mathrm{ult}}-0.5\right)}^{2}\right]}^{0.4},V/V_{\mathrm{ult}}>0.50\\ T/T_{\mathrm{ult}}=1,V/V_{\mathrm{ult}}\leq 0.50 \end{array}$$

(8) $$\begin{array}{c} V/V_{\mathrm{ult}}=0.5+0.5{\left[1-{\left(T/T_{\mathrm{ult}}\right)}^{2.5}\right]}^{0.3},V/V_{\mathrm{ult}}>0.50\\ T/T_{\mathrm{ult}}\;=1,V/V_{\mathrm{ult}}\leq 0.50 \end{array}$$

(9) $$\begin{array}{c} V/V_{\mathrm{ult}}=0.4+0.6\sqrt{1-{\left(T/T_{\mathrm{ult}}\right)}^{3.5}},V/V_{\mathrm{ult}}>0.40\\ T/T_{\mathrm{ult}}\;=1,V/V_{\mathrm{ult}}\leq 0.40 \end{array}$$

### Moment-horizontal loading

**Figure 11.**
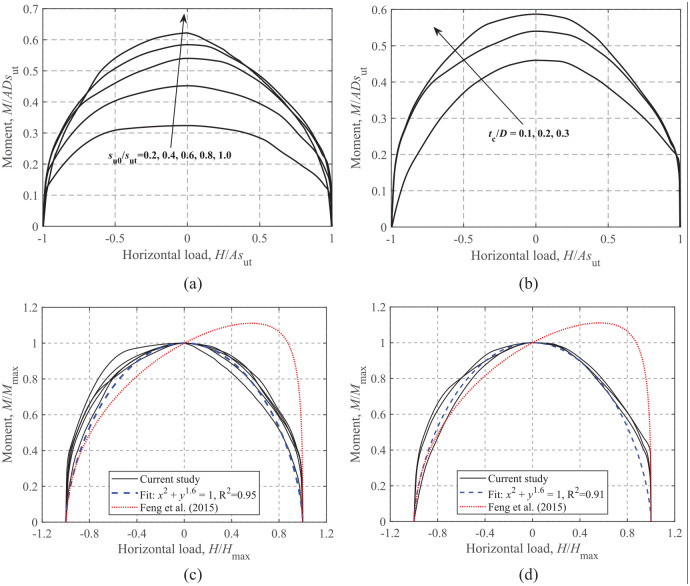
*M*-*H* failure envelopes at V/V_ult_ = 0.50: (a) dimensionless, t_c_/D = 0.2, (b) dimensionless, s_u0_/*s*_ut_ = 0.6, (c) normalized, t_c_/D = 0.2, and (d) normalized, s_u0_/*s*_ut_ = 0.6.

The effects of *s*_u0_/*s*_ut_ and *t*_c_/*D* on the *M-H* failure envelopes are illustrated in Figure 11. As the failure envelopes for V/V_ult_ = 0.25, 0.50, and 0.75 are similar in shape (only sizes are different), only the results for V/V_ult_ = 0.50 are presented for brevity. The equation proposed by Feng et al. (2015) for rectangular foundations on a soil with a crust under an unlimited-tension interface condition is also compared. Figure 11a and b show that with the increase of *s*_u0_/*s*_ut_ and *t*_c_/*D*, an expansion of the failure envelopes is observed, but a similar shape of the curves is expressed. This feature may assist in eliminating the dependence on *s*_u0_/*s*_ut_ and *t*_c_/*D* levels by normalizing the failure envelopes by their corresponding maximum values, as shown in Figure 11c and d. Moreover, the current *M-H* curves are almost symmetrical about *H* = 0, while the curve proposed by Feng et al. (2015) is oblique due to the effect of the unlimited-tension interface. In this study, a unique equation, expressed as:

(10) $${\left(H/H_{\max}\right)}^{2}+{\left(M/M_{\max}\right)}^{1.6}=1$$

is used to simulate the normalized envelopes. The comparison shows that this simple expression provides reasonable fits for various *s*_u0_/*s*_ut_ and *t*_c_/*D* levels.

### Horizontal-torsional loading

**Figure 12.**
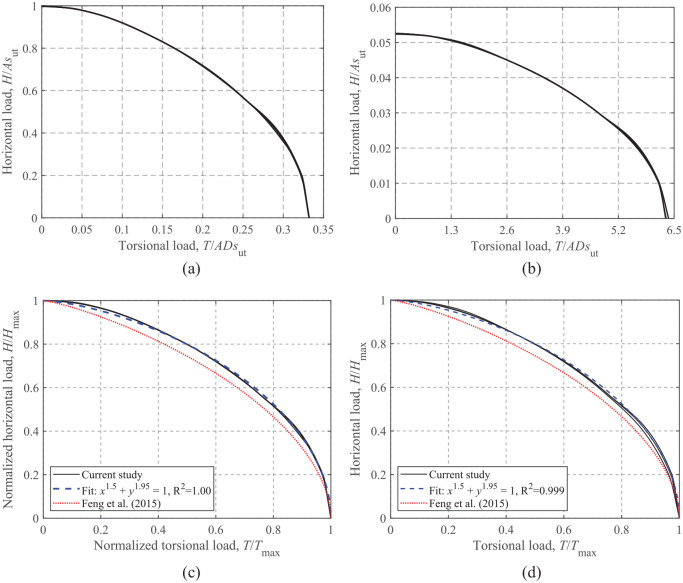
*H*-*T* failure envelopes at V/V_ult_ = 0.50: (a) dimensionless, t_c_/D = 0.2, (b) dimensionless, s_u0_/*s*_ut_ = 0.6, (c) normalized, t_c_/D = 0.2, and (d) normalized, s_u0_/*s*_ut_ = 0.6.

Similar to the *M-H* envelopes shown in Figures 11 and 12 shows the effects of *s*_u0_/*s*_ut_ and *t*_c_/*D* on the *H-T* failure envelopes at V/V_ult_ = 0.50. The dimensionless envelopes shown in Figure 12a and b exhibit a high similarity regardless of the values of *s*_u0_/*s*_ut_ and *t*_c_/*D*. As shown in Figure 12c and d, the equation proposed by Feng et al. (2015) lies slightly inside the normalized envelopes. In addition, equation (11) proposed by Finnie and Morgan (2004) is considered to fit the *H-T* failure envelopes. The dimensionless powers, *l* = 1.5 and *n* = 1.95, in equation (11) yield satisfactory fits, as can be seen from Figure 12c and d.

(11) $${\left(H/H_{\max}\right)}^{l}+{\left(T/T_{\max}\right)}^{n}=1$$

### Moment-torsional loading

**Figure 13.**
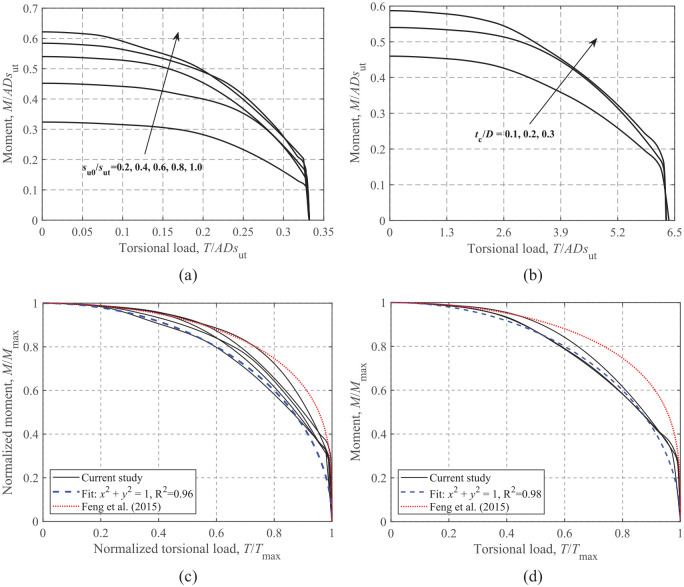
*M*-*T* failure envelopes at V/V_ult_ = 0.50: (a) dimensionless, t_c_/D = 0.2, (b) dimensionless, s_u0_/*s*_ut_ = 0.6, (c) normalized, t_c_/D = 0.2, and (d) normalized, s_u0_/*s*_ut_ = 0.6.

The effects of *s*_u0_/*s*_ut_ and *t*_c_/*D* on the ultimate load-carrying capacity under moment and torsional loading at V/V_ult_ = 0.50 are shown in Figure 13. As shown in Figure 13a and b, the *M-T* envelope expands with increasing *s*_u0_/*s*_ut_ due to the increase of the maximum moment. Normalization by the corresponding maximum moment and torsional loads also leads to a relatively narrow band of the curves. The comparison shows that the expression given by Feng et al. (2015) seems to be slightly unconservative. The analytical relationship of equation (12) with the two dimensionless parameters equal to 2 is compared to the FE results in Figure 13c and d, where favorable predictions are observed.

(12) $${\left(M/M_{\max}\right)}^{\alpha }+{\left(T/T_{\max}\right)}^{\beta }=1$$

## Failure envelopes under combined VHMT loadings

This section derives an analytical expression for the 4-D failure envelope in VHMT loading space. Three sets of notations are defined: (1) $V_{\mathrm{ult}}$ , $H_{\mathrm{ult}}$ , $M_{\mathrm{ult}}$ , $T_{\mathrm{ult}}$ —uniaxial ultimate capacities; (2) $H_{\max}$ , $M_{\max}$ , $T_{\max}$ —maximum capacities at a given level of the vertical load without the other load components; and (3) $H_{\max}\prime$ , $M_{\max}\prime$ —reduced maximum capacities at a given level of the vertical load with a non-zero torsional load (*T* ≠ 0).

Based on the above notations and the forms of equation used in the previous sections, the general forms of the calculated envelopes are summarized in equations (13)–(15). $f_{h}$ , $f_{m}$ and $f_{t}$ are functions of the vertical load level. Specific expressions for these failure envelopes can be found in the previous sections.

(13) $$\frac{H_{\max}}{H_{\mathrm{ult}}}=f_{h}\left(\frac{V}{V_{\mathrm{ult}}}\right),\frac{M_{\max}}{M_{\mathrm{ult}}}=f_{m}\left(\frac{V}{V_{\mathrm{ult}}}\right),\frac{T_{\max}}{T_{\mathrm{ult}}}=f_{t}\left(\frac{V}{V_{\mathrm{ult}}}\right)$$

(14) $${\left(\frac{H}{H_{\max}}\right)}^{a}+{\left(\frac{M}{M_{\max}}\right)}^{b}=1$$

(15) $${\left(\frac{H_{\max}^{\prime }}{H_{\max}}\right)}^{c}+{\left(\frac{T}{T_{\max}}\right)}^{d}=1,{\left(\frac{M_{\max}^{\prime }}{M_{\max}}\right)}^{e}+{\left(\frac{T}{T_{\max}}\right)}^{f}=1$$

Equation (14), which describes the *M-H* failure envelope under the condition of *T* = 0, is taken as the basic function. However, a more generalized equation for the *M-H* failure envelope under the condition of *T* ≠ 0 is required to derive the final VHMT envelope expression. Due to the very similar shape of the *M-H* failure envelope (only the sizes are different), it is reasonable to assume that under the condition of *T* ≠ 0, equation (14) is still applicable for the *M-H* failure envelope normalized by the corresponding maximum values, $H_{\max}\prime$ and $M_{\max}\prime$ (reduce to $H_{\max}$ and $M_{\max}$ in equation (14) if *T* = 0). Therefore, equation (14) can be replaced by a more generalized form:

(16) $${\left(\frac{H}{H_{\max}^{\prime }}\right)}^{a}+{\left(\frac{M}{M_{\max}^{\prime }}\right)}^{b}=1$$

Mathematical manipulations of equations (13), (15), and (16) allow the formulation of an analytical expression for the failure envelope in VHMT loading space in terms of $V/V_{\mathrm{ult}}$ , $H/H_{\mathrm{ult}}$ , $M/M_{\mathrm{ult}}$ , and $T/T_{\mathrm{ult}}$ :

(17) $$f\left(\frac{V}{V_{\mathrm{ult}}},\frac{H}{H_{\mathrm{ult}}},\frac{M}{M_{\mathrm{ult}}},\frac{T}{T_{\mathrm{ult}}}\right)={\left(\frac{H/H_{\mathrm{ult}}}{{\left[1-{\left(\frac{T/T_{\mathrm{ult}}}{f_{t}\left(V/V_{\mathrm{ult}}\right)}\right)}^{d}\right]}^{\frac{1}{c}}\cdot f_{h}\left(V/V_{\mathrm{ult}}\right)}\right)}^{a}+{\left(\frac{M/M_{\mathrm{ult}}}{{\left[1-{\left(\frac{T/T_{\mathrm{ult}}}{f_{t}\left(V/V_{\mathrm{ult}}\right)}\right)}^{f}\right]}^{\frac{1}{e}}\cdot f_{m}\left(V/V_{\mathrm{ult}}\right)}\right)}^{b}=1$$

In practical design, the design loads (factored loads and materials), VHMT, can be directly substituted into the left-hand side of equation (17); values less than 1 represent a sufficient ultimate limit design and vice versa.

**Figure 14.**
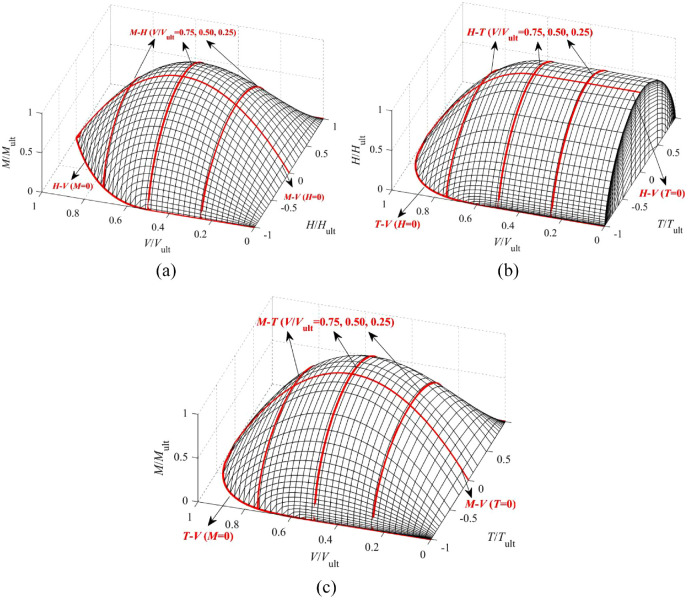
Normalized 3-D failure surfaces (He and Newson, 2022a, 2022b): (a) VHM at *T* = 0, (b) VHT at *M* = 0, and (c) VMT at *H* = 0.

To visualize the shape of the 4-D normalized failure surface, three 3-D failure surfaces in terms of $V/V_{\mathrm{ult}}$ , $H/H_{\mathrm{ult}}$ , $M/M_{\mathrm{ult}}$ , and $T/T_{\mathrm{ult}}$ are presented in Figure 14 (He and Newson, 2022a). The specific curves obtained from the FE results are also incorporated for comparison. For the VHT and VMT failure surfaces, the portion of *T*<0 is presented considering the symmetry about the plane of *T* = 0.

## Application for design

**Figure 15.**
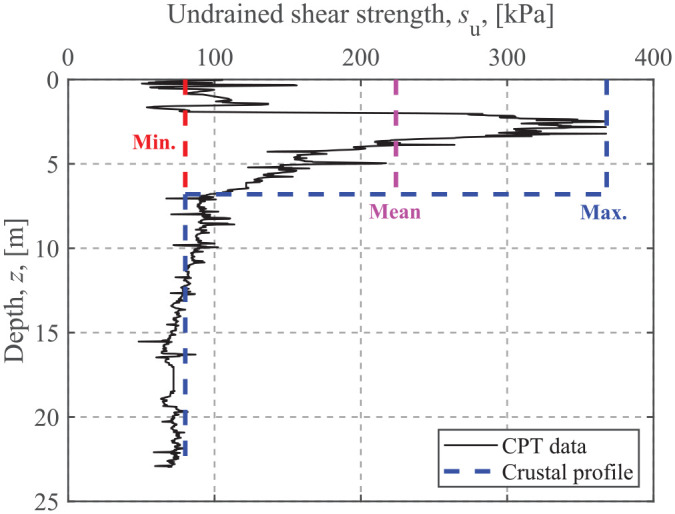
Undrained shear strength profile.

As an example of the application of the method, the ultimate limit design of a shallow foundation of a typical onshore wind turbine is addressed using the failure envelope method. The diameter of the surface circular foundation is 19 m, and the undrained shear strength estimated with a cone penetration test (CPT) is shown in Figure 15. Due to the existence of fissuring and stress-strain compatibility, the high undrained shear strength of the upper crust is unlikely to be fully mobilized at failure (Lefebvre et al., 1987). Rochelle et al. (1974) recommended to use the full (i.e. maximum strength value), mid-depth (i.e. strength value at mid-depth of the crust), and minimum (i.e. strength value just below the crust) strength of the upper crust for limit state analysis. For the *s*_u_ profile shown in Figure 15, the mid-depth strength is close to the maximum value. Lefebvre et al. (1987) adopted the mean value of the maximum strength of the upper crust and the minimum strength of the underlying soil. In this analysis, the undrained shear strength of the underlying soil is held constant at *s*_u0_ = 80 kPa, and the minimum (*s*_ut_ = 80 kPa), maximum (*s*_ut_ = 368 kPa), and mean (*s*_ut_ = 224 kPa) undrained shear strength of the crust are adopted for comparison. The minimum strength case reduces the soil to a single-layer material. The crust thickness is assumed to be *t*_c_ = 6.8 m. The assumed undrained shear strength profiles are shown in Figure 15. In addition, the factored ultimate limit state loads from International Electrotechnical Commission (2005) are *V* = 24,900 kN, *H* = 1100 kN, *M* = 76,200 kN × m and *T* = 4400 kN × m.

**Figure 16.**
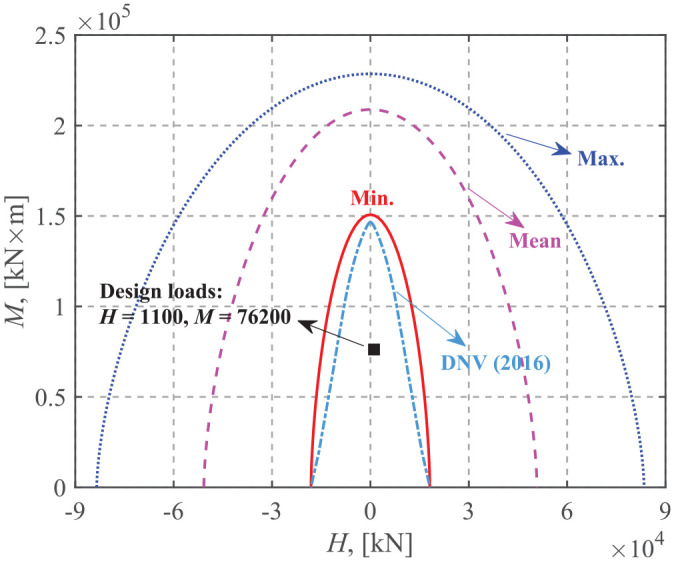
Design loads compared to *M-H* failure envelopes.

The corresponding uniaxial bearing capacities are computed based on the factored undrained shear strength (as recommended by DNV, GL, 2016, partial safety factor for materials, *γ*_m_ = 1.25 for total stress analysis), as summarized in Table 2. The safety check for the ultimate limit design using the failure envelope method shows that the three cases yield acceptable ultimate capacity designs. However, the soil with a surficial crust gains extra margins of safety compared to the single-layer one. Figure 16 shows the comparison between the reduced *M-H* failure envelopes and the factored design loads. These *M-H* envelopes are obtained by substituting the factored design vertical and torsional loads into equation (17) in order to incorporate the effects of vertical and torsional loads. For comparison, the traditional approach using the load inclination factor and the effective foundation area (DNV, GL, 2016) is also incorporated for the case of a single-layer soil. It can be seen that the traditional approach is more conservative than the failure envelope method, which has also been confirmed by Shen et al. (2016). In addition, the considerable difference in the absolute sizes of the three failure envelopes indicates that ignoring the contribution of the upper crust may significantly underestimate the bearing capacity and lead to a great overdesign of the foundation.

**Table 2.** Ultimate limit state design of the foundation.

Soil profile	Ultimate capacity	Normalized load	Safety check
Min. (i.e. single-layer)	*V*_ult_ = 108,875 [kN]	*V*/*V*_ult_ = 0.229	$f\left(\frac{V}{V_{\mathrm{ult}}},\frac{H}{H_{\mathrm{ult}}},\frac{M}{M_{\mathrm{ult}}},\frac{T}{T_{\mathrm{ult}}}\right)$ = 0.340 < 1 (Safe!)
	H_ult_ = 18146 [kN]	*H*/*H*_ult_ = 0.061	
	*M*_ult_ = 213,758 [kN·m]	*M*/*M*_ult_ = 0.357	
	*T*_ult_ = 113,774 [kN·m]	*T*/*T*_ult_ = 0.039	
Mean	*V*_ult_ = 214,226 [kN]	*V*/*V*_ult_ = 0.116	$f\left(\frac{V}{V_{\mathrm{ult}}},\frac{H}{H_{\mathrm{ult}}},\frac{M}{M_{\mathrm{ult}}},\frac{T}{T_{\mathrm{ult}}}\right)$ = 0.200 < 1 (Safe!)
	*H*_ult_ = 50,808 [kN]	*H*/*H*_ult_ = 0.022	
	*M*_ult_ = 508,307 [kN·m]	*M*/*M*_ult_ = 0.150	
	*T*_ult_ = 318,568 [kN·m]	*T*/*T*_ult_ = 0.014	
Max.	*V*_ult_ = 285,771 [kN]	*V*/*V*_ult_ = 0.087	$f\left(\frac{V}{V_{\mathrm{ult}}},\frac{H}{H_{\mathrm{ult}}},\frac{M}{M_{\mathrm{ult}}},\frac{T}{T_{\mathrm{ult}}}\right)$ = 0.173 < 1 (Safe!)
	*H*_ult_ = 83,471 [kN]	*H*/*H*_ult_ = 0.013	
	*M*_ult_ = 718,420 [kN·m]	*M*/*M*_ult_ = 0.106	
	*T*_ult_ = 523,362 [kN·m]	*T*/*T*_ult_ = 0.0084	

## Conclusions

The VHMT failure envelopes of circular shallow foundations resting on a stiff crust which overlies the main soil deposit under undrained soil conditions have been investigated using finite element analysis. A zero-tension interface condition was considered. Five values of *s*_u0_/*s*_ut_ and three values of *t*_c_/*D* were used to investigate the effects of *s*_u0_/*s*_ut_ and *t*_c_/*D* on the failure envelopes. For the uniaxial vertical and moment capacities, crust correction factors have been introduced to account for the effects of *s*_u0_/*s*_ut_ and *t*_c_/*D*. Analytical equations of the crust correction factors, which are functions of *s*_u0_/*s*_ut_ and *t*_c_/*D*, were also proposed. The same forms of equation that are used for uniform soils, but with different parameters provide good fits for the VHMT failure envelopes for a soil with a surficial crust. To facilitate the application of the failure envelope method in practical foundation design, an analytical expression for the 4-D VHMT failure envelope was derived based on the FE results. An example application of the use of this method for design was also presented. The analysis indicated that neglecting the contribution of the crustal soil layer may significantly underestimate the bearing capacity of the foundation. These approaches should aid the assessment of the ultimate capacity of shallow foundations under complex loading conditions.
